# Amorphous ITZO-Based Selector Device for Memristor Crossbar Array

**DOI:** 10.3390/mi14030506

**Published:** 2023-02-22

**Authors:** Ki Han Kim, Min-Jae Seo, Byung Chul Jang

**Affiliations:** 1 School of Electronic and Electrical Engineering, Kyungpook National University, 80 Daehakro, Bukgu, Daegu 41566, Republic of Korea; 2 Department of Electronic Engineering, Gachon University, 1342 Seongnam-daero, Seongnam 13120, Republic of Korea

**Keywords:** memristor crossbar array, amorphous In-Sn-Zn-O (a-ITZO), selector device

## Abstract

In the era of digital transformation, a memristor and memristive circuit can provide an advanced computer architecture that efficiently processes a vast quantity of data. With the unique characteristic of memristor, a memristive crossbar array has been utilized for realization of nonvolatile memory, logic-in-memory circuit, and neuromorphic system. However, the crossbar array architecture suffers from leakage of current, known as the sneak current, which causes a cross-talk interference problem between adjacent memristor devices, leading to an unavoidable operational error and high power consumption. Here, we present an amorphous In-Sn-Zn-O (a-ITZO) oxide semiconductor-based selector device to address the sneak current issue. The a-ITZO-selector device is realized with the back-to-back Schottky diode with nonlinear current-voltage (I-V) characteristics. Its nonlinearity is dependent on the oxygen plasma treatment process which can suppress the surface electron accumulation layer arising on the a-ITZO surface. The a-ITZO-selector device shows reliable characteristics against electrical stress and high temperature. In addition, the selector device allows for a stable read margin over 1 Mbit of memristor crossbar array. The findings may offer a feasible solution for the development of a high-density memristor crossbar array.

## 1. Introduction

Digital transformation (DT) has accelerated the exponential expansion of total digital data volume. In particular, the global pandemic has influenced individuals to not only work remotely from home, study, and engage in e-commerce, but also to actively embrace Internet of Things (IoT) technology. This trend has caused a paradigm shift from processor-centric computing to memory-centric computing. To keep up with the new paradigm, a vast volume of data must be efficiently processed on the edge device. Memristor and memristive circuit are one of the candidates for the advanced computing architecture which differs from the conventional von Neumann architecture suffering from data movement between the central processing unit and memory. The memristor has been widely developed for promising nonvolatile memory and artificial synapse device due to its simple two-terminal structure, fast switching speed, and low power consumption [1,2,3,4,5]. In addition, memristive circuit can implement a nonvolatile logic-in-memory circuit that enables normally off computing [6,7,8,9].

The memristor crossbar array is the optimal architecture for enabling high packing density, defect tolerance, vector-matrix product operation, and logic-in-memory operation [10,11,12]. During memristor operation, however, this architecture generates undesired leakage currents, known as sneak currents, which flow through unselected neighboring devices during memristor operation. These sneak currents lead to the operational error, high power consumption, poor performance of memristive neuromorphic system, and the limited logic operations in only a row of device [9,13], hence restricting the maximum memristor array size. Vertical integration of a memristor device and a two-terminal selector device with nonlinear current–voltage (I-V) characteristics is required to tackle these issues. The selector device requires a symmetric I-V nonlinearity for bipolar switching memristor, which suppresses the current flow at a low-voltage region while permitting a much greater current (about >100) in a high-voltage region [14]. Numerous researchers have scrutinized various two-terminal selector devices, which include the threshold switching device [14,15,16,17], 0T2M structure [18], mixed-ionic conductor device [19], multilayer tunneling device [14], and the back-to-back Schottky diode [20,21,22]. The threshold switching based on the metal-to-insulator (MIT) transition of VO_2_ [15] and the fast relaxation of Ag conductive filament [17] suffer from the poor thermal stability because the VO_2_ is a well-known material occurring at the MIT transition at a low temperature of 340 K [23] and the accelerated relaxation of the Ag conductive filament by high temperature. Furthermore, although the 0T2M structure is effective to suppress the sneak current, the larger area of 0T2M is not suitable for high-density memristor crossbar array. Therefore, the back-to-back Schottky diode is preferred for a stackable high-density memristor array due to its ease of fabrication, low thermal budget, and simple structure design. Although there has been research on back-to-back Schottky diodes, Ni/TiO2/Ni devices have demonstrated superior performance [24,25]. However, the TiO_2_-based selector device requires well-controlled stoichiometry, crystallinity of the TiO_2_, and insufficient current density by the poor carrier mobility of this intrinsic material.

Amorphous-oxide-semiconductor (AOS) thin films have received significant attention as active layers in driving thin-film transistors (TFTs) in the display industry due to their inherent amorphous properties enabling high device-to-device uniformity, low manufacturing cost, and high transparency by virtue of its wide bandgap (3 eV) [26,27]. Although the amorphous In-Ga-Zn-O (a-IGZO) material is currently utilized in the display industry, an amorphous In-Sn-Zn-O (a-ITZO) material is suitable for selector device applications due to its higher electron mobility (20–30 cm^2^/Vs) compared to a-IGZO [28]. Nevertheless, a stable Schottky barrier formation with this material is challenging, because the surface electron accumulation layer (SEAL) naturally arises from the oxide semiconductor (OS) surface reduced by formation of oxygen vacancy in a vacuum chamber [29,30,31,32]. Deposition of a conductive polymer [33], a chemical surface treatment [34,35], and oxygen plasma treatment [31,32,36,37] have all been proposed to impede and remove the formation of SEAL. Among these strategies, oxygen plasma treatment is suitable to develop the OS-based selector device because it can provide a uniform and reliable Schottky barrier with a high oxidation rate at low temperatures. Therefore, it is necessary to investigate the oxygen plasma treated a-ITZO-based selector in order to develop a high-performance engineering methodology.

In this study, we investigate the a-ITZO-based back-to-back Schottky diode type selector device. Depending on the oxygen plasma treatment process conditions, the a-ITZO-selector device demonstrated a clear elimination of SEAL with the improved nonlinear I-V characteristics. With the optimized oxygen plasma process, the selector device with Pd/a-ITZO/Pd structure showed a symmetric nonlinearity of I-V, reliable endurance, bias stress, and temperature stabilities. In addition, the simulation results for calculating reading margin with bipolar memristor revealed that the integrated memristor array with the a-ITZO-selector is capable of achieving a maximum array size of more than 1 Mbit. Therefore, these results provide not only a-ITZO-selector device is suitable for memristive crossbar array, but also the engineering strategy to improve the performance of the a-ITZO-selector, as well as other OS-based selector devices.

## 2. Materials and Methods

The a-ITZO-selector devices with the structure of Pd/a-ITZO/Pd were fabricated with the crossbar architecture. In this device, the Pd with high work function (5.22 eV) [38] is adopted for both top and bottom electrode to form a Schottky barrier with n-type semiconductor a-ITZO which has 4.5 eV of electron affinity. First, 60 nm-thick Pd electrodes were deposited SiO_2_/Si substrate using thermal evaporation method through a shadow mask. Then, a 30 nm thick a-ITZO film was deposited on the bottom Pd electrodes using radio-frequency (RF) sputtering under high vacuum conductions (0.088 Pa). The atomic ratio of the deposited a-ITZO film is investigated using X-ray photoelectron spectroscopy depth profile (Figure S1). The atomic ratio of a-ITZO film without oxygen plasma treatment has In:Sn:Zn:O = 22:4:18:56. After that, the oxygen plasma using an inductively coupled plasma asher system was treated to remove the SEAL in a-ITZO film. Finally, 60 nm thick Pd top electrodes were deposited perpendicular to the Pd bottom electrodes using the thermal evaporation method. Both line widths of Pd were identical at 60 μm. The fabricated a-ITZO-selector device was electrically characterized by providing a voltage to the Pd TE while the Pd BE was grounded using a Keithley 4200 semiconductor parameter analyzer in an air environment. To evaluate the performance of a-ITZO-selector for suppression of sneak current in the memristor crossbar array, we performed the numerical simulation through MATLAB using the experimental values for the LRS/HRS resistances and SET/RESET voltages of TiO_2_-based bipolar switching memristor and the resistance of a-ITZO-selector. The TiO_2_-memristor with the structure of AI/a-TiO_2_/AI was fabricated using amorphous titanium oxide films which are deposited by plasma enhanced atomic layer (PEALD) deposition method (ASM Genitech MP-1000) [39]. Titanium tetraisopropoxide (TTIP) and oxygen plasma were used as precursors for Ti and oxygen, respectively. For a-TiO2 film deposition utilizing PEALD, 100 cycles of ALD at 80 °C were performed. The cycle was comprised of TTIP (2 s), Ar (4 s), O_2_ gas (2 s), and Ar (2 s). The radio frequency (RF) pulse was applied for 1.5 s with 150 W of RF power during the injection of O_2_ gas. The Al bottom electrode was deposited by thermal evaporation through a metal shadow mask and then an a-TiO_2_ film was formed on the Al bottom electrode. The top Al electrode was deposited using thermal evaporation method, which forms a crossbar array structure.

## 3. Results and Discussion

**Figure 1:**
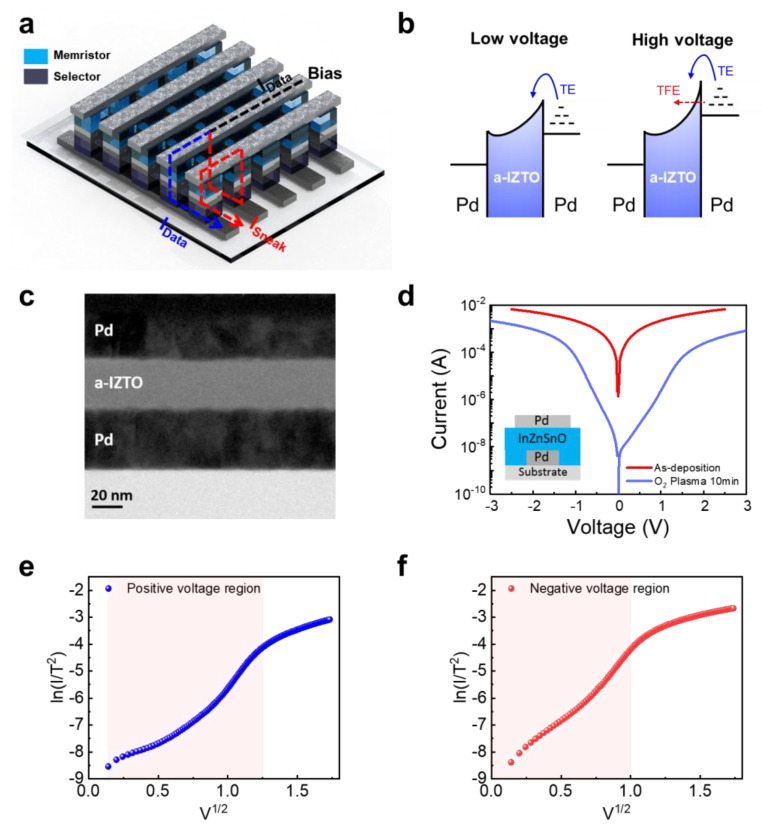
(**a**) Schematic of the sneak current path in memristor crossbar array during operation. (**b**) Band diagram and conduction mechanism of back-to-back Schottky diode under low- and high-voltage regions. (**c**) Cross-sectional HRTEM image of the a-ITZO-selector device. (**d**) I–V characteristics of a-ITZO-selector device before and after O_2_ plasma treatment prior to deposition of top Pd electrode. The results of the I-V characteristics are fitted by thermionic emission in (**e**) the positive-voltage region and (**f**) the negative-voltage region. The shaded red regions are the thermionic emission region where the current increases exponentially with the voltage on a linear scale.

The sneak current path in the memristor crossbar array is illustrated in a schematic format in Figure 1a, which depicts the operating process. If there is no selector device, the sneak currents will flow through the devices in the surrounding region that have not been selected, as shown by the red arrow line. This will result in an error in the operation of the memristor crossbar array. To suppress the sneak current, we developed the back-to-back Schottky diode with a Pd/a-ITZO/Pd structure on SiO_2_/Si substrate. The Schottky diode should operate the thermionic emission over the Schottky barrier while both thermionic emission and thermionic field emission across the Schottky barrier are dominated in the high-voltage region, as shown in Figure 1b. Note that the conduction mechanism in the low-voltage region only works with thermionic emission and is not dominated by tunneling current across the Schottky barrier to suppress the sneak current in the memristor crossbar array by increasing nonlinearity of the Schottky diode-based selector device. This results in a high level of nonlinearity of I-V characteristics. Generally, tunneling current across the Schottky barrier happens when the width of the Schottky barrier is reduced as a result of a high doping concentration. It is essential to inhibit the formation of oxygen vacancy in OS material, also known as dopant, in order to achieve the high nonlinearity of I-V that is achieved by suppressing the tunneling component. This will allow for the achievement of high nonlinearity of I-V. Therefore, we employed Pd, which is a noble metal with a high work function (5.22 eV) [38] when compared to the electron affinity of a-ITZO (4.5 eV).

This is due to the fact that the noble Pd electrode has a very high Gibbs free energy of oxide formation.

The fabricated a-ITZO-selector device is shown in Figure 1c as a cross-sectional high-resolution transmission electron microscopy (HRTEM) image. It was confirmed that an a-ITZO layer with a thickness of 30 nm was formed uniformly between the top and bottom Pd electrodes. The as-deposited a-ITZO-selector device showed the almost ohmic behavior, which indicates the formation of SEAL (Figure S2). In contrast, the a-ITZO-selector devices, which had an oxygen plasma treatment applied to the surface of the a-ITZO before the top Pd electrode was deposited, showed nonlinear I-V characteristics, as shown in Figure 1d. The I-V characteristics of the oxygen plasma-treated a-ITZO-selector can be fitted by the thermionic emission equation ($ln\left(\frac{I}{T^{2}}\right)\propto \;V^{0.5})$. Figure 1e,f shows the currents in both the low-voltage and high-voltage regions, where the currents are exponentially proportional to voltage on a linear scale, are well fitted to the thermionic emission, indicating that thermionic emission is the dominant conduction mechanism.

These characteristics indicate the suppression of current flow in low-voltage regions of both positive and negative voltage while permitting a larger current flow in either direction in high-voltage regions. These nonlinear I-V characteristics demonstrate that the oxygen plasma treatment is an effective method for Schottky barrier formation by eliminating SEAL.

**Figure 2:**
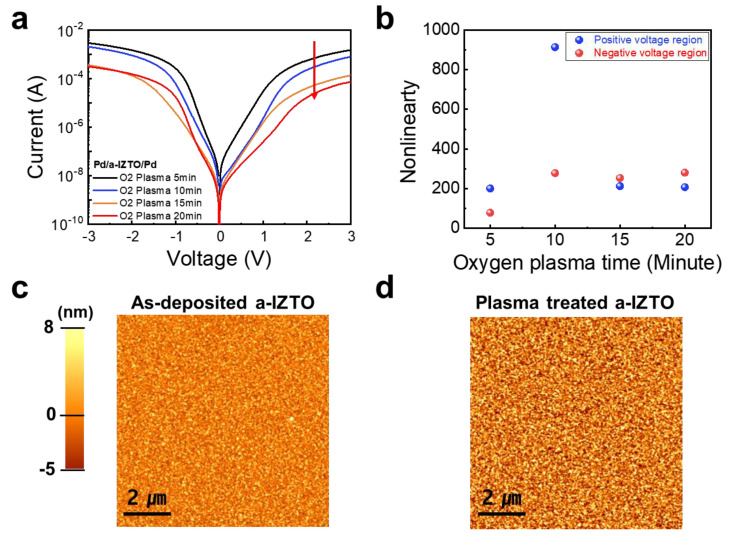
(**a**) I-V characteristics of a-ITZO-selector device depending on the process time of oxygen plasma treatment. As shown in the red arrow, the current decreases as the O_2_ plasma time increases. (**b**) Nonlinearity value of a-ITZO-selector device as a function of oxygen plasma process time. AMF image of a-ITZO surface (**c**) before and (**d**) after oxygen plasma treatment.

The oxygen plasma treatment process is the essential technology to form the Schottky barrier of a-ITZO material. We investigated the effect that the oxygen plasma treatment had on the a-ITZO-selector device by analyzing the electrical properties of an a-ITZO-selector device to determine how they changed as a function of the time that the device was exposed to oxygen plasma. Considering that the RF power of oxygen plasma can affect the penetration depth of the oxygen radical, a plasma process with a power more than 100 W is capable of oxidizing the surface region of a-ITZO. This oxidation of a-ITZO with the harsh oxygen plasma treatment results in an increase in the resistivity of its selector device, which can result in a degradation of the nonlinearity of its I-V characteristics. We adopted the 100 W of oxygen plasma treatment process to get rid of SEAL solely on the surface of a-ITZO. When the oxygen plasma treatment condition is maintained for more than 15 min, the current in the high-voltage region decreases rapidly. On the other hand, the currents in the positive low-voltage region and negative-voltage region decrease abruptly at the 15 min and the 20 min of oxygen plasma treatment, respectively (Figure 2a). Figure 2b exhibits the nonlinearities of I-V characteristics which are extracted from the current ratio at *V*_Read_ (±1.8 V) and *V*_Read_/3 (±0.6 V) in both the positive- and negative-voltage regions. The memristor crossbar array can be operated with the V/2, V/3, and ground schemes [40]. The optimal bias scheme depends on the selectivity of the selector device and the operating SET/RESET voltages of the memristor. Considering that we developed the a-ITZO-selector device for TiO_2_-based memristor array, the nonlinearity was evaluated using the V/3. Among the times of oxygen plasma treatment processes, the a-ITZO-selector device with the oxygen treatment time of 10 min exhibited the best nonlinearity value of 914 and 280 for the positive voltage and negative voltage regions, respectively. The worst value of about 70 of the nonlinearity is attributed to the partial removal of SEAL by the insufficient oxygen plasma treatment. On the other hand, the degraded nonlinearity caused by harsh oxygen plasma treatment for more than 15 min results from a significant decrease in current level in the high-voltage region, which is caused by the suppression of thermionic field emission by the reduced completely oxygen vacancy. In addition, in our previous study, we analyzed the chemical composition of a-ITZO film with oxygen plasma treatment using XPS [41]. It was observed that the O 1s core level at 531.9 eV related to O-O bonding absorbed on the a-ITZO surface increases by oxygen plasma treatment. Other research groups have also reported that oxygen adsorbed on the oxide semiconductor surface eliminates the SEAL [37,42]. These findings indicate that the naturally formed SEAL can be eliminated using the optimal oxygen plasma. An atomic force microscopy (AFM) investigation was carried out so that the effect of the oxygen plasma treatment that was applied to the a-ITZO surface could be investigated in further detail. The AFM topographic images of the a-ITZO film before and after being treated with oxygen plasma are represented in Figure 2c, and d, respectively. When comparing the surface roughness of 2.6 nm after oxygen plasma treatment for 10 min with the surface roughness of 2.4 nm before oxygen treatment, it was confirmed that 10 min of oxygen plasma did not significantly affect the microstructure/topology of the a-ITZO material surface. In addition, the surface roughness is 2.6 nm and 2.8 nm for 15 min and 20 min of oxygen plasma treatment at 100 W, respectively (Figure S3). The electrical characteristics and AFM analyses indicate that the optimized oxygen plasma treatment technique is capable of forming a Schottky barrier in the a-ITZO-selector device without damaging the microstructure/topology of surface of a-ITZO material.

**Figure 3:**
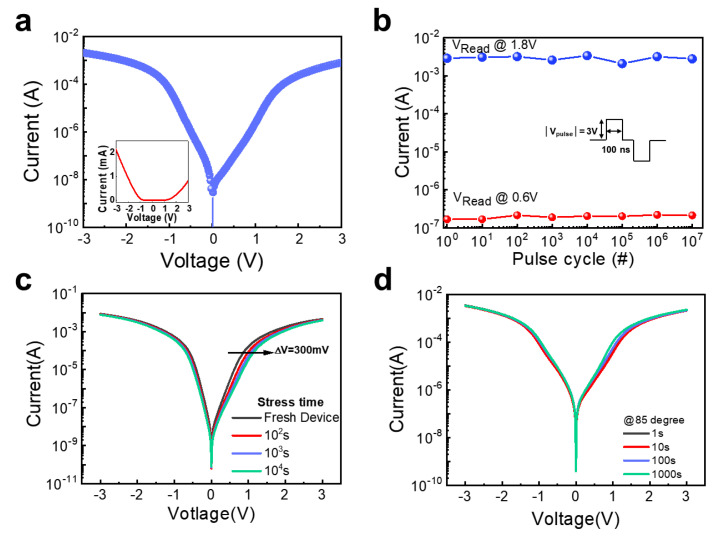
(**a**) I-V characteristics of oxygen plasma treated a-ITZO-selector device. The inset shows the linear I-V plot. Electrical stability tests under (**b**) cycling endurance and (**c**) constant bias stress. (**d**) Temperature stability test.

To investigate the electrical stability of the a-ITZO-selector device fabricated through oxygen plasma treatment for 10 min at 100 W, cycling endurance, and constant bias stability tests were performed. Figure 3a exhibits a representative I-V characteristic of the a-ITZO-selector device. As shown in the inset of Figure 3a, the linear I-V plot indicates the apparent rectifying characteristics that result from the formation of a Schottky barrier. The results of the cycle endurance test that was carried out under the repeated voltage pulses are shown in Figure 3b. This test was carried out to validate the stability of the Schottky barrier that was formed utilizing the oxygen plasma treatment. In this cycling endurance test, voltage pulses with 100 ns width and ±3 V amplitude were applied over 10^7^ cycles. While the repeated pulse voltages were being applied, the currents at the read voltages *V*_Read_ (1.8 V) and *V*_Read_/3 (0.6 V) were being measured. The currents at *V*_Read_ and *V*_Read_/3 was sustained without significant degradation, indicating that the nonlinearity of I-V characteristics was preserved. The constant bias stability test was conducted for 10^4^ s under a constant voltage bias in order to further validate the stability of Schottky barrier (Figure 3c). The constant bias instability test was performed by applying a constant voltage of 3 V until a certain time, which is the same as the general bias instability test of tin-film transistor. Because of electron trapping at the Pd/a-ITZO interface, the I-V curves of the a-ITZO-selector device shifted toward the positive voltage region by 300 mV when subjected to a constant bias stress, as shown in Figure 3c. However, the nonlinearity of I-V characteristics was maintained without significant degradation, indicating the stable formation of the Schottky barrier. In addition, in order to investigate the temperature stability of the formed Schottky barrier, we performed the stability test at the temperature of 85 °C. As shown in Figure 3d, although the a-ITZO-selector device was tested for 1000 s at the temperature of 85 °C, the selector device did not experience a significant degradation. As electrical and temperature stability of the selector device are critical requirements, these results indicate that the oxygen plasma treated a-ITZO-selector device can be suitable for a high-density memristor application.

**Figure 4:**
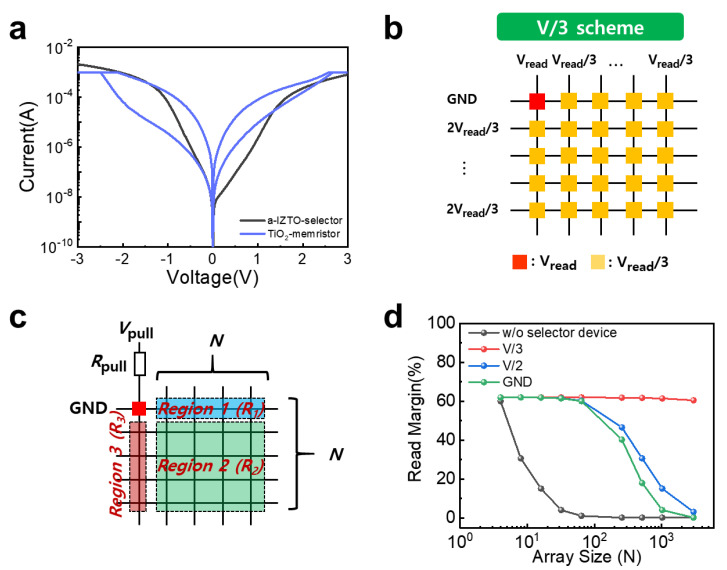
(**a**) I-V characteristics of TiO_2_-memristor and a-ITZO-selector device. (**b**) V/3 scheme for operation of the memristor crossbar array. (**c**) Equivalent circuit of an N X N memristor crossbar array to calculate the reading margin. (**d**) Calculated reading margin according to ground, V/2, and V/3 schemes as a function of array size.

To evaluate the performance of the memristor crossbar integrated a-ITZO selector device, the read margin, which is related to the maximum size of the memristor array, was evaluated. To investigate the read margin, we utilized the TiO_2_-based memristor with a structure of Al/TiO_2_/AI which shows bipolar resistive switching [39] (Figure 4a). Due to the modification of the interfacial AI-Ti-O layer by oxygen ion migration, the TiO_2_-based memristor operates with the positive-voltage region with the SET process and in the negative-voltage region with the RESET process. The TiO_2_-based memristor is suitable for calculating the read margin, which can be improved by a-ITZO-selector, as the SET and RESET voltages of TiO_2_-meristor are in the voltage region where the high current flows in nonlinearity of a-ITZO-selector. For the reading operation of the memristor device in the crossbar array, ground, V/2, and V/3 reading bias schemes were generally utilized. In the ground scheme, all the unselected top and bottom lines were grounded. The *V*_Read_ voltages are only applied to the devices with a selected top line, where most sneak currents are generated. All unselected top and bottom lines were biased at half the read voltage (*V*_Read_/2) in the V/2 scheme, permitting sneak currents to flow through the half-selected devices in the selected top and bottom lines. In the V/3 scheme, all the unselected top and bottom lines were biased at one-third (*V*_Read_/3) and two-thirds (2*V*_Read_/3) of the read voltage, generating sneak currents across the selected devices with the voltage drop magnitude of *V*_Read_/3, with the exception of the selected cells in the crossbar array, as depicted in Figure 4b. In the numerical calculation, the worst-case scenario (all unselected cells with low resistance state) was considered, and the three reading bias schemes were assessed to determine the optimal reading bias scheme. MATLAB was utilized to obtain the numerical solution to the reading margin computation. The maximum crossbar array size was determined using a 10% reading margin as the criterion. To access the information stored in the selected device, a pull-up voltage (*V_pull_*) through pull-up resistance (*R_pull_*) is applied to the selected top line, while the selected bottom line is grounded. Depending on the reading scheme, the regions of the unselected devices can be categorized as Region 1, Region 2, and Region 3, which can be expressed by three resistor networks as depicted in Figure 4c. For ground scheme, the applied voltages of Region 1 and Region 2 are ground, whereas the applied voltage of Region 3 is *V*_Read_. For V/2 scheme, the applied voltages for Region 1 and Region 3 are *V*_Read_/2, while for V/3 scheme, the applied voltages for all regions are *V*_Read_/3. We determined the reading margin based on the above three resistor networks by solving the following equation:

$$R.\;M.=\frac{V_{out,HRS}-V_{out,LRS}}{V_{pull}}=\frac{R\left(HRS\right)∥\left(R_{1}+R_{2}+R_{3}\right)}{R_{pull}+R\left(HRS\right)∥\left(R_{1}+R_{2}+R_{3}\right)}-\frac{R\left(LRS\right)∥\left(R_{1}+R_{2}+R_{3}\right)}{R_{pull}+R\left(LRS\right)∥\left(R_{1}+R_{2}+R_{3}\right)}$$

where $V_{out,HRS}$ is the voltage drop at the output node caused by the selected device in a high-resistance state (HRS), whereas $V_{out,LRS}$ is the voltage drop at the output node caused by the selected device in a low-resistance state (LRS). We calculated the reading margin using the LRS/HRS resistances and SET/RESET voltages of TiO_2_-memristor and the resistance of a-ITZO -selector. It was confirmed that the ground scheme has the maximum reading margin (Figure 4d). In contrast, the V/2 and V/3 schemes show the reading margin degradation as the array size increases. The obvious differences in reading margin among the three reading schemes are attributed to the source of the sneak currents. The ground scheme generates leakage current only from the devices with the selected top line, while the other two reading schemes induce leakage currents from half and one-third of the selected devices on the memristor crossbar array. In addition, the sneak current degrades the SET voltage window which is defined by the difference between the SET voltage delivered to the selected cell and the maximum voltage applied to the most disturbed unselected cell [43]. Thanks to the selector device, the TiO_2_-memristor shows the only about 25% degradation of the SET voltage window, which is calculated using numerical simulation through MATLAB [43] (Figure S4). It is noteworthy that the size of the memristor array with integrated a-ITZO-selector device can be expanded to 1 Mbit by utilizing two V/2 and V/3 methods.

## 4. Conclusions

In conclusion, we have demonstrated the feasibility of a-ITZO-selector device for a memristor crossbar array. To develop the back-to-back Schottky diode type selector device based on a-ITZO material, we employed an oxygen plasma treatment prior to Pd top electrode deposition in order to eliminate SEAL. The effect of time condition on the oxygen plasma treatment process for the a-ITZO-selector was explored, revealing that optimizing the process time is crucial for attaining nonlinear I-V characteristics. The a-ITZO-selector device that has been treated with oxygen plasma exhibits nonlinear I-V characteristics, stable cycling endurance, constant bias stability, and temperature stability. In addition, to prove the practical application of selector devices, the integrated TiO_2_-memristor and a-ITZO-selector device array demonstrates that its memristor array size can rise to 1 Mbit in density. Compared to the state-of-the-art selector device for memristor crossbar array, the a-ITZO-selector device showed the comparable reliability against electrical stress. It is noted that the a-ITZO-selector device exhibited temperature stability at 85 °C, thanks to the intrinsic material property of an oxide semiconductor. Other selector devices including threshold switching based on MIT transition and the fast relaxation of Ag filament suffer from the poor temperature stability by their intrinsic material property. Therefore, we believe that these results on the a-ITZO-selector device can give a viable approach for realizing a high-density, reliable memristor array.

## Data Availability

Not applicable.

## Supplementary Materials

The following supporting information can be downloaded at: https://www.mdpi.com/article/10.3390/mi14030506/s1, Figure S1: XPS depth profile result of the deposited a-ITZO film; Figure S2: Linear plot of I-V characteristics of a-ITZO-selector with and without oxygen plasma treatment; Figure S3: AMF image of oxygen plasma traeted a-ITZO film with (a) 15 min and (d) 20 min at 100 W; Figure S4: Calculated SET voltage window with and without selector device as a function of array size.
