# Supplementary material for: Amorphous ITZO-Based Selector Device for Memristor Crossbar Array
Source: Micromachines (Basel). 2023 Feb 22;14(3):506. doi: 10.3390/mi14030506 (PMC10054342; doi:10.3390/mi14030506)
Supplement: Supplementary file 1 [file micromachines-14-00506-s001.zip › micromachines-2205368-supplementary.pdf]

Supporting Information for

Amorphous ITZO-based selector device for memristor crossbar array

Ki Han Kim [1], Min-Jae Seo [2,*] and Byung Chul Jang [1,3,*]

[1]School of Electronic and Electrical Engineering, Kyungpook National University, 80 Daehakro, Bukgu, Daegu41566, Republic of Korea

[2]Department of Electronic Engineering, Gachon University, 1342 Seongnam-daero, Seongnam13120, Republic of Korea

[3]School of Electronic and Electrical Engineering, Kyungpook National University, 80 Daehakro, Bukgu, Daegu41566, Republic of Korea

*Authors to whom correspondence should be addressed.
Correspondence: bc.jang@knu.ac.kr, mjseo@gachon.ac.kr

Supplemenatry Information

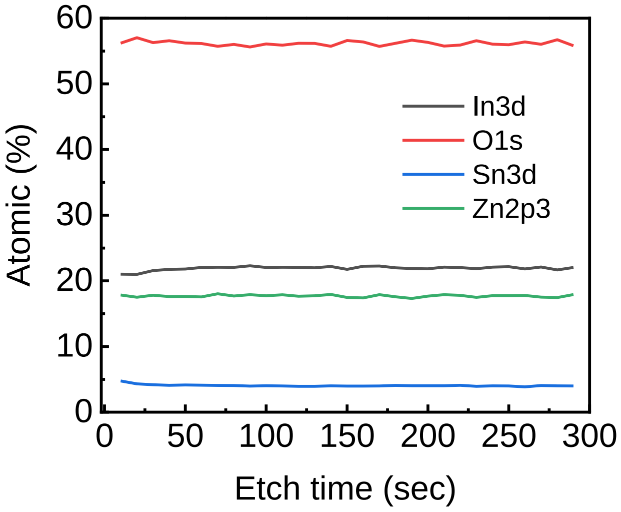

Figure S1. XPS depth profile result of the deposited a-ITZO film.

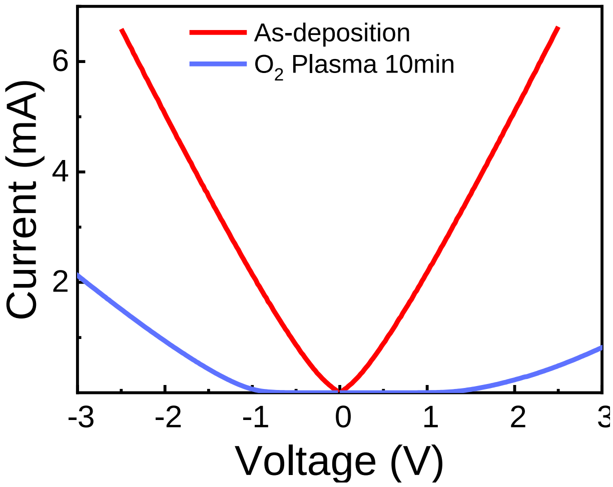

Figure S2. Linear plot of I-V characteristics of a-ITZO-selector with and without oxygen plasma treatment.

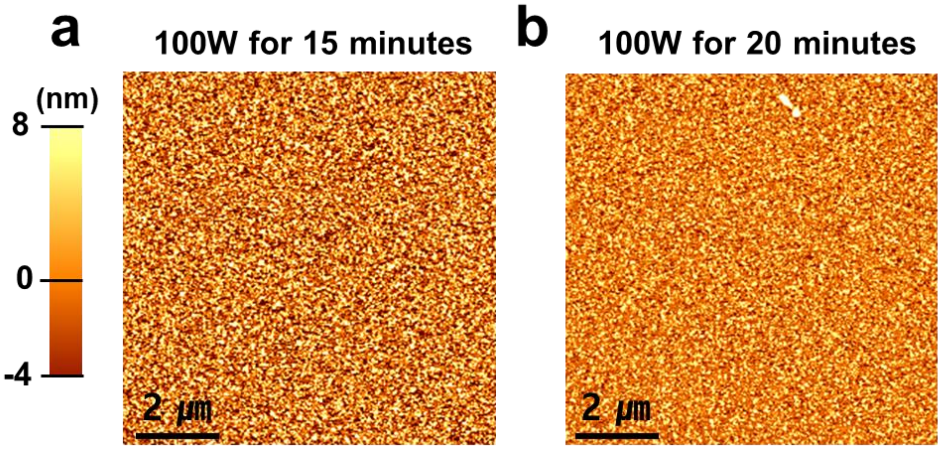

Figure S3. AMF image of oxygen plasma traeted a-ITZO film with (a) 15 minutes and (d) 20 minutes at 100 W.

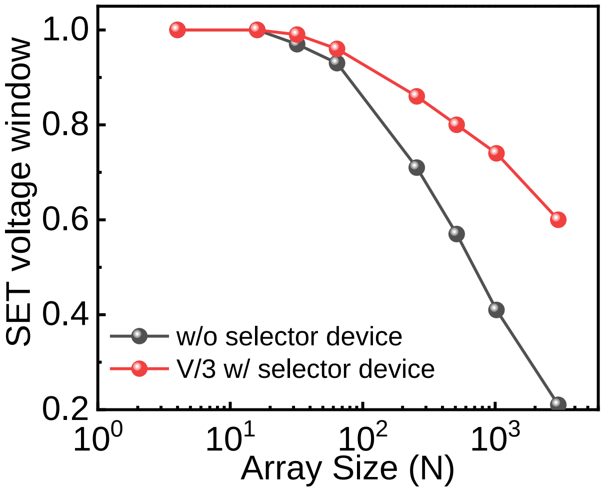

Figure S4. Calculated SET voltage window with and without selector device as a function of array size.
